# Effects of anti-inflammatory agents on cardiovascular outcomes: A systematic review and meta-analysis of randomised controlled trials

**DOI:** 10.1016/j.ajpc.2026.101642

**Published:** 2026-04-17

**Authors:** A Perkovic, ER Webster, PM Ridker, KR Tuttle, V Perkovic, BL Neuen

**Affiliations:** a School of Medicine and Public Health, University of Newcastle, Newcastle, Australia; b The Children’s Hospital at Westmead, Sydney, Australia; c Center for Cardiovascular Disease Prevention, Brigham and Women’s Hospital, Harvard Medical School, Boston MA, USA; d Cardiovascular Division, Brigham and Women’s Hospital, Harvard Medical School, Boston MA, USA; e Nephrology Division, University of Washington School of Medicine, Seattle WA, USA; f Providence Inland Northwest Health, Spokane WA, USA; g University of New South Wales, Sydney, Australia; h Department of Renal Medicine, Royal North Shore Hospital, Sydney, Australia; i The George Institute for Global Health, University of New South Wales, Sydney, Australia

**Keywords:** Inflammation, Cardiovascular outcomes, Infection, Cancer

## Abstract

**Background:**

Inflammation is causally implicated in the development and progression of atherosclerotic cardiovascular disease, but clinical trials of anti-inflammatory therapies have yielded inconsistent effects on cardiovascular outcomes.

**Methods:**

We conducted a systematic review and meta-analysis to evaluate the cardiovascular efficacy and safety of a broad range of anti-inflammatory agents. Medline, Embase and Cochrane databases were searched from inception to 08 October 2024 for randomised controlled trials evaluating the effect of anti-inflammatory therapies on a primary cardiovascular outcome with at least 100 patient-years follow-up per treatment arm. Trial level meta-analysis was performed using a random effects model. The primary outcome was major adverse cardiovascular events (MACE); other outcomes included myocardial infarction, stroke, heart failure, serious adverse events, infection, and malignancy.

**Results:**

Thirteen trials enrolling 82,208 participants were included. The effect of anti-inflammatory agents on MACE varied by drug class (P-heterogeneity=0.049), driven primarily by benefits observed with colchicine (RR 0.76; 95 % CI 0.65–0.90; moderate certainty) and canakinumab (RR 0.88; 95 % CI 0.79–0.97; moderate certainty), with no benefit observed for other agents. Among colchicine trials, heterogeneity was identified (P-heterogeneity=0.003), and subgroup analyses suggested greater benefit in coronary artery disease and/or recent myocardial infarction trials (P-heterogeneity=0.068). Safety outcomes also varied by drug class, with significant heterogeneity in serious adverse events (P-heterogeneity=0.005), largely attributable to methotrexate, and some evidence of heterogeneity for infection (P-heterogeneity=0.076) and malignancy (P-heterogeneity=0.077).

**Conclusion:**

Some anti-inflammatory agents may reduce the risk of cardiovascular outcomes, but their effects appear to vary substantially across drug classes, with important differences in both efficacy and safety. These findings underscore the need for rigorous evaluation of new anti-inflammatory therapeutics to ascertain benefits and harms across different populations and therapeutic indications.

## 1 Introduction

Inflammation plays an important role in the development and progression of atherosclerotic cardiovascular disease [1]. Observational and Mendelian randomisation analyses indicate that systemic inflammation, reflected in high levels of high sensitivity C-reactive protein (CRP) and/or interleukin-6 (IL-6), is associated with increased risk of cardiovascular events [[1], [2], [3]].

Based on the results of several randomized trials, colchicine in 2023 became the first anti-inflammatory agent approved by the United States Food and Drug Administration to reduce risk of major adverse cardiovascular events (MACE) in people with established cardiovascular disease or multiple risk factors [4]. However, several subsequent large outcome trials of colchicine have not demonstrated cardiovascular benefits [[5], [6], [7], [8]]. Furthermore, the results of trials assessing the effects of other anti-inflammatory agents have been mixed [9,10]. It is not clear whether these differences are related to chance, study power, populations, therapeutic indications, or differences across therapeutic agents. Both infection and malignancy have been reported as adverse effects of some anti-inflammatory agents, but again it is unclear whether these reflect differences in the various drug classes or other factors [10,11].

Understanding the therapeutic landscape of agents that reduce inflammation may provide important insights for ongoing efforts to develop safe and effective therapies that target anti-inflammatory pathways to improve cardiovascular outcomes. We therefore conducted a systematic review and meta-analysis to synthesise evidence on the effects of a broad range of anti-inflammatory agents on cardiovascular as well safety outcomes, including infections and malignancy.

## 2 Methods

This systematic review and meta-analysis was conducted and reported according to the Preferred Reporting Items for Systematic reviews and Meta-Analyses (PRISMA) statement and prospectively registered on PROSPERO (CRD42021237087).

### 2.1 Search strategy

We performed a systematic search of Medline, Embase and Cochrane databases without language restriction from database inception up to 08 October 2024 for randomised controlled trials evaluating the effects of anti-inflammatory agents, specifically, methotrexate, colchicine, interleukin-1 inhibitors (canakinumab, anakinra, rilonacept), interleukin-6 inhibitors (toclizumab, sarilumab, ziltivekimab), JAK-1/2 inhibitors (baricitinib), NRF-2 activators (bardoxolone), ASK-1 inhibitors (selonsertib), PKC-alpha inhibitors (ruboxistaurin), CCL-2 inhibitors (CCX140-B), TNF alpha inhibitors (etanercept), phospholipase A2 inhibitors (darapladib, varespladib), briakinumab and cenicriviroc. References of select review articles were screened for additional eligible studies. The broad inclusion of mechanistically diverse anti-inflammatory agents was designed to provide a comprehensive basis for comparing and contrasting cardiovascular effects across therapeutic classes. Details of the database search strategy are displayed in Table S1.

### 2.2 Study selection and outcomes

We restricted inclusion to randomized active- or placebo-controlled trials evaluating anti-inflammatory therapies on a primary cardiovascular outcome, with ≥100 patient-years of follow-up per arm. This approach ensured sufficient exposure time to accrue cardiovascular events and prevented the inclusion of trials primarily designed to assess non-cardiovascular outcomes. The exact definition of the primary outcome was allowed to vary across trials. We excluded trials of corticosteroids and non-steroidal anti-inflammatory drugs due to their wide range of actions and indications that are unlikely to be used for cardiovascular risk reduction. We excluded trials in which the anti-inflammatory agents were used for specific immunosuppressive or malignancy related indications, for example, prevention of graft versus host disease or gestational trophoblastic disease, and those where there was use of anti-inflammatory agents in all study arms. For long term extension studies, only the original trial data were assessed.

### 2.3 Outcomes

The primary outcome was MACE, defined as nonfatal myocardial infarction, nonfatal stroke or cardiovascular death. To maximise use of available trial-level data, we adopted the MACE definition used within each individual trial. For trials that did not report a strict three-point MACE, this typically reflected composite outcomes that included additional components such as coronary revascularisation or hospitalisation for unstable angina, or substituted all-cause mortality for cardiovascular mortality. Other cardiovascular and mortality outcomes included: myocardial infarction, stroke, heart failure, cardiovascular death and all-cause death. We also evaluated a range of adverse outcomes including serious adverse events, infections, and cancer.

### 2.4 Data extraction and risk of bias assessment

Two study authors (AP, EW) independently screened all trials for eligibility and extracted data using a standardised data extraction software, with any discrepancies resolved in consultation with a third author (VP). Data on trial design, population, interventions, and relevant outcomes in each trial were recorded. Risk of bias was evaluated using version 2 of the Cochrane risk of bias tool for randomised trials (RoB 2) [12]. Potential publication bias was assessed by visual inspection of funnel plots of log-transformed hazard ratios against their standard errors. Formal statistical tests for funnel plot asymmetry were not performed given the limited number of included trials. Authors were contacted for further data where relevant outcomes could not be obtained from published reports.

### 2.5 Data synthesis and analysis

We prespecified that treatment effects on cardiovascular and safety outcomes were to be quantitatively synthesised using a random effects model to obtain summary treatment effects estimates expressed as relative risks with associated 95 % confidence intervals (95 % CI). We pooled, in order of preference, hazards ratios, incidence rate ratios, and risk ratios (based on the number of events and participants) to maximise the information obtained from trial-level data. Between study variation was evaluated based on I^2^ and P heterogeneity values obtained from a random effects model. For all outcomes, we conducted subgroup analyses by major drug class with p values for heterogeneity across subgroups obtained from the same random effects model.

We summarized the certainty of evidence for the primary outcome using the Grading of Recommendations Assessment, Development, and Evaluation (GRADE) approach based on within-study risk of bias, indirectness of evidence, unexplained heterogeneity or inconsistency of results, and imprecision of results. Because of the significant heterogeneity observed across drug classes for the primary outcome, we performed separate GRADE assessments for each individual drug class. We evaluated the robustness of the effect of anti-inflammatory agents on the primary outcome in sensitivity analyses excluding non-coronary artery disease or post myocardial infarction trials (e.g., stroke prevention trials), and trials in which all-cause death was substitute for cardiovascular death.

Because we observed evidence of benefit but substantial heterogeneity across colchicine trials – and given that colchicine is the only anti-inflammatory agent currently approved for cardiovascular risk reduction – we conducted additional post-hoc subgroup analyses restricted to the colchicine trials. Subgroups were defined by age (<65 vs ≥65 years), sex, diabetes status, history of coronary revascularization, estimated glomerular filtration rate (<60 vs ≥60 mL/min/1.73m²), follow-up duration (<1 year vs ≥1 year), and trial population (coronary artery disease/post-myocardial infarction vs non–coronary artery disease).

All analyses were performed using Stata, version 18.0 [13].

## 3 Results

We identified 10,353 records in the database search with 9186 articles remaining after duplicates were removed (Figure S1). After screening articles by title and abstract, we assessed 478 full text articles for eligibility and included 122 reports of 13 randomised trials. These included 8 trials of colchicine [[5], [6], [7], [8],^11^,[14], [15], [16]], 2 trials of darapladib [17,18], and one trial of each of canakinumab [9], methotrexate [10] and varespladib [19]. 12 trials enrolled patients with established cardiovascular disease and 1 trial assessed a primary cardiovascular outcome in patients undergoing non-cardiac surgery (Table 1). Median trial duration ranged from 0.5 to 48 months and study size varied from 532 to 15,828 participants (Table 1).

**Table 1:** Characteristics of included studies.

Trial	Setting	Population	Duration (months)	Participants	Intervention	Primary outcome	Mean Age	Female
VISTA-16	Multicentre (International)	Acute coronary syndrome	4	5145	Varespladib	Major adverse cardiovascular events	61	26
LoDoCo	Australia	Coronary disease	36	532	Colchicine	Major adverse cardiovascular events	66	11
LoDoCo2	Multicentre (International)	Coronary disease	29	5522	Colchicine	Major adverse cardiovascular events	66	15
SOLID-TIMI 52	Multicentre (International)	Acute coronary syndrome	30	13,026	Darapladib	Major adverse cardiovascular events	64	74
CANTOS	Multicentre (International)	Prior myocardial infarction	48	10,061	Canakinumab	Major adverse cardiovascular events	61	26
CIRT	Multicentre (North America)	Coronary disease, type 2 diabetes	28	4786	Methotrexate	Major adverse cardiovascular events	66	19
COLCOT	Multicentre (International)	Prior myocardial infarction	23	4745	Colchicine	Major adverse cardiovascular events	61	19
COPS	Multicentre (Australia)	Coronary disease	12	795	Colchicine	Major adverse cardiovascular events	60	21
STABILITY	Multicentre (International)	Coronary disease	44	15,828	Darapladib	Major adverse cardiovascular events	65	19
CHANCE-3	Multicentre (China)	Ischaemic stroke or transient ischaemic attack	3	8343	Colchicine	Stroke	66	38
CLEAR-SYNERGY	Multicentre (International)	Myocardial infarction with percutaneous coronary intervention	34	7062	Colchicine	Major adverse cardiovascular events	61	20
CONVINCE	Multicentre (International)	Ischaemic stroke or transient ischaemic attack	36	3154	Colchicine	Major adverse cardiovascular events	66	30
COP-AF	Multicentre (International)	Non-cardiac thoracic surgery	0.5	3209	Colchicine	Atrial fibrillation or myocardial injury	68	48

Risk of bias was variable across trials (Figure S2). Of 13 included trials, nine were at low risk of bias overall, four were moderate risk and none were deemed high risk. Visual inspection of the funnel plot did not demonstrate clear or substantial asymmetry. While some dispersion was observed among smaller studies, there was no obvious absence of small studies in areas of non-significance (Figure S3).

### 3.1 Cardiovascular and mortality outcomes

**Fig. 1:**
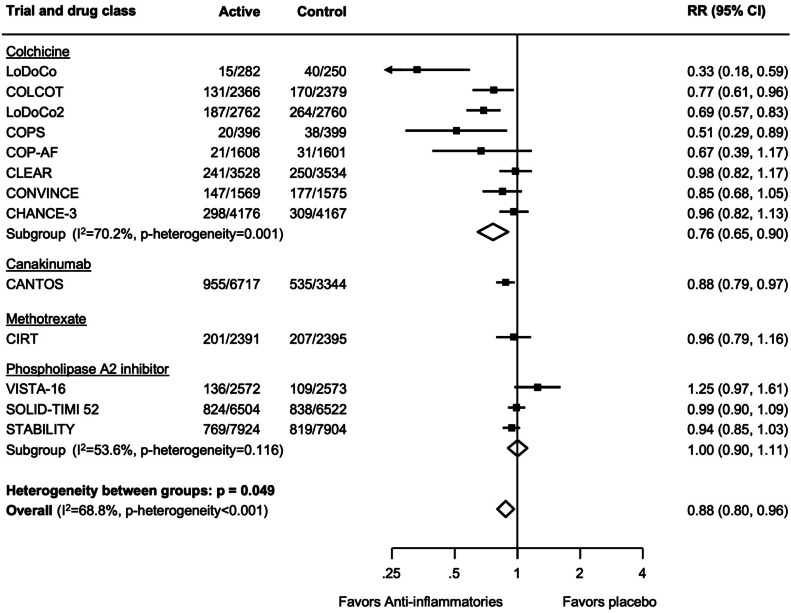
Effect of anti-inflammatory agents on major adverse cardiovascular outcomes.

Anti-inflammatory therapy reduced the incidence of MACE overall (RR 0.88, 95 % CI 0.80–0.96; Fig. 1). However, there was significant heterogeneity across trials (I^2^=68.8 %; P-heterogeneity<0.001) and drug classes (P-heterogeneity 0.049). Benefits on the primary outcome were driven primarily by colchicine (RR 0.76, 95 % CI 0.65–0.90; moderate certainty) and canakinumab (RR 0.88, 95 % CI 0.79–0.98; moderate certainty), with no clear benefit observed for other anti-inflammatory agents (moderate certainty for methotrexate and high certainty for phospholipase A2 inhibitors). A sensitivity analysis excluding non-coronary artery disease or post-myocardial infarction trials yielded similar results overall (RR 0.87, 95 % CI 0.78–0.98), with significant heterogeneity again observed across trials (I^2^=75.3 %; P-heterogeneity<0.001) and drug classes (P-heterogeneity=0.045; Figure S4). The findings from an additional sensitivity analysis excluding trials that included all-cause death as part of the MACE composite outcome were unchanged overall (RR 0.90, 95 % CI 0.82–0.98), although the test for heterogeneity across drug classes was no longer statistically significant (P-heterogeneity=0.12; Figure S5).

**Fig. 2:**
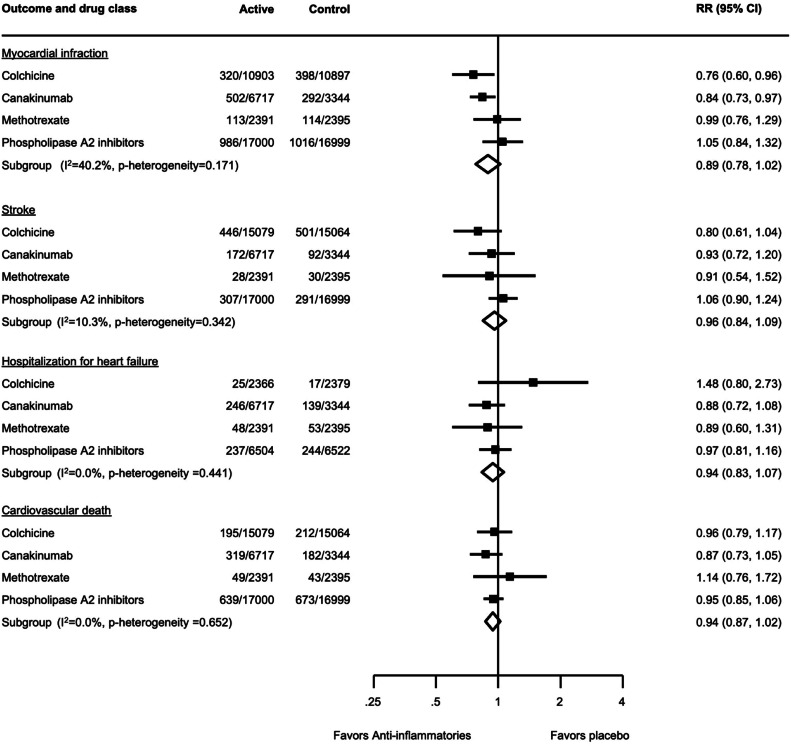
Effect of anti-inflammatory agents on myocardial infarction, stroke, hospitalization for heart failure, and cardiovascular death, overall and by drug class.

**Fig. 3:**
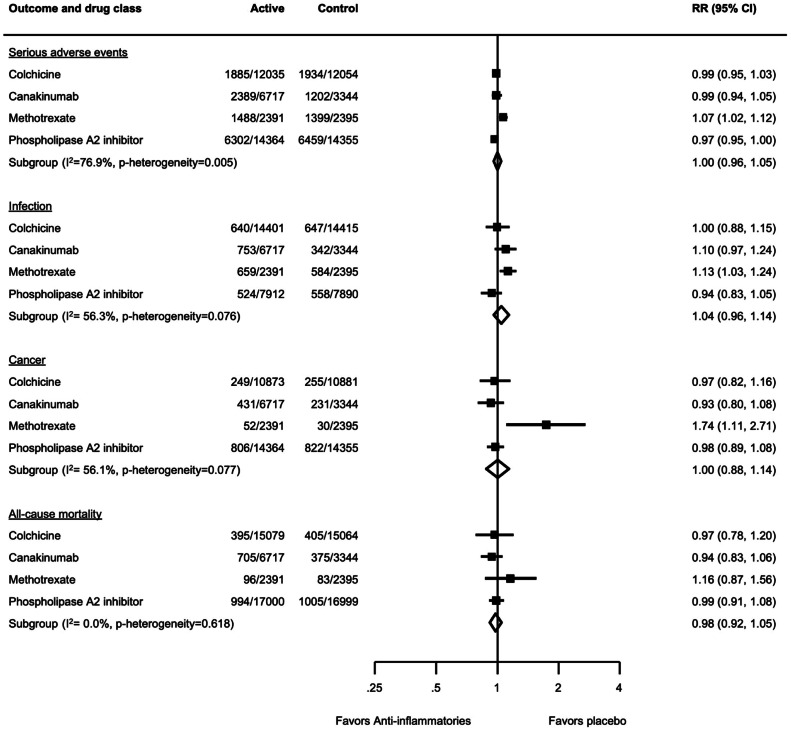
Effect of anti-inflammatory agents on serious adverse events, infection, cancer, and all-cause mortality, overall and by drug class.

The effects of anti-inflammatory agents on individual cardiovascular outcomes are displayed in Fig. 2. Anti-inflammatory agents did not reduce the risk of any cardiovascular outcome, with no statistical evidence of heterogeneity across agents for myocardial infarction (I^2^=40.2 %; P-heterogeneity=0.17), stroke (I^2^=10.3 %; P-heterogeneity=0.34), hospitalization for heart failure (I^2^=0 %, P-heterogeneity=0.44), or cardiovascular death (I^2^=0 %; P-heterogeneity=0.65). Reductions in myocardial infarction were observed for colchicine (RR 0.76, 95 % CI 0.60–0.96) and canakinumab (RR 0.84, 95 % CI 0.73–0.97), but not for other drug classes. No reductions in hospitalization for heart failure hospitalisation were observed for any anti-inflammatory drug class (Fig. 2). No clear effect was observed for cardiovascular death or all-cause death overall (Fig. 2, Fig. 3). Effects on cardiovascular and mortality outcomes by individual trials are summarised in Figures S6–9.

### 3.2 Safety outcomes

The effects of anti-inflammatory agents on key safety outcomes are displayed in Fig. 3. Although no effect on serious adverse events was observed overall (RR 1.00, 95 % CI 0.96–1.05), there was significant heterogeneity across drug classes (I^2^=76.9 %, P-heterogeneity=0.005) with an increased risk identified for methotrexate (RR 1.07, 95 % CI 1.02–1.12), but not other therapeutic agents. Similarly, while no clear effects increased risk of infection was observed overall, there was significant heterogeneity across drug classes (I^2^=56.3 %; P-heterogeneity=0.08) with methotrexate associated with an increased risk of infection (RR 1.13, 95 % CI 1.03–1.24). The same pattern of effect was observed for cancer (I^2^=56.1 %; P-heterogeneity=0.077), driven by an increase risk of non-basal cell skin cancer with methotrexate (RR 1.74, 95 % CI 1.11–2.71). Effects on safety outcomes by individual trial are summarised in Figures S11–13.

### 3.3 Subgroup analyses of colchicine trials

**Fig. 4:**
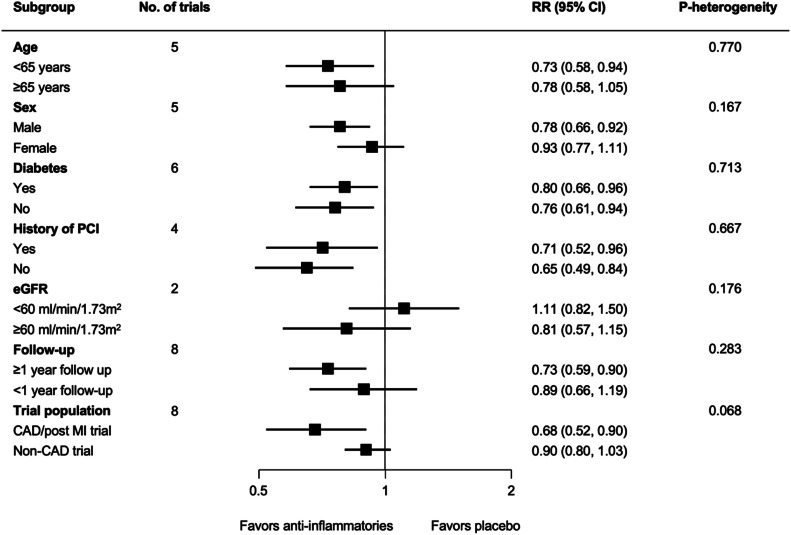
Subgroup analysis of the effect of colchicine on major adverse cardiovascular events.

The effect of colchicine on the primary outcome varied significantly across trials (I^2^=70.2 %; P-heterogeneity=0.003; Fig. 1). In post-hoc subgroup analyses restricted to the colchicine trials, treatment effects on MACE were generally consistent across subgroups defined by age, sex, diabetes status, history of coronary revascularization, estimated glomerular filtration rate, and median follow-up duration (all P-heterogeneity > 0.167; Fig. 4). However, there was some evidence that the magnitude of benefit was greater in the coronary artery disease or recent myocardial infarction trials (P-heterogeneity = 0.068).

## 4 Discussion

This systematic review and meta-analysis provides an updated and comprehensive assessment of the cardiovascular efficacy and safety of a broad range of anti-inflammatory therapies. The effect of anti-inflammatory therapy on cardiovascular outcomes was highly heterogenous across drug classes, with the most consistent evidence of benefit observed for colchicine and canakinumab. The significant heterogeneity even within drug classes - including among colchicine trials – highlights that cardiovascular efficacy and safety are not uniform across agents or populations. Aside from clear differences in the mechanism of action of individual therapeutic agents, population differences across these trials – including demographic data, duration of therapy and history of cardiovascular disease – may have contributed to the heterogeneity observed. These findings underscore the importance of rigorous evaluation of emerging anti-inflammatory therapies to clarify their benefits and risks across diverse clinical settings and populations.

In 2023, the United States Food and Drug Administration approved colchicine to reduce MACE in patients with, or at high risk of atherosclerotic cardiovascular disease [4]. However, the results of subsequent trials of colchicine have been mixed, and adoption in routine clinical practice is limited. Notably, no benefit of colchicine was observed in the more recent CLEAR trial which studied 7062 patients with recent myocardial infarction [6]. Along with the beneficial effect of canakinumab in reducing cardiovascular events in the CANTOS trial, these data have provided key randomised evidence that targeting inflammation can reduce the risk of atherosclerotic cardiovascular events. However, our review highlights the complexity of the therapeutic landscape for reducing systemic inflammation, with significant heterogeneity across multiple outcomes for a broader range of anti-inflammatory agents.

Colchicine and canakinumab, the two therapies that have been shown to have atherosclerotic cardiovascular benefits, both inhibit IL-1β, a key molecule in stimulating CRP production [7,20,21]. In contrast, other agents, such as methotrexate and ruboxistaurin, act on inflammatory pathways that have neither reduced CRP in prior studies nor provided cardiovascular protection [10,[22], [23], [24]]. Baricitinib has been demonstrated to reduce CRP though the effects of this drug class have not been evaluated in dedicated cardiovascular outcome trials [25]. Thus available data suggest that targeting IL-1β, TNFα, IL-6, and CRP pathways may be most effective for addressing residual cardiovascular risk [26]. Studies evaluating IL-6 inhibition and cardiovascular outcomes were not available for inclusion for this review but are being studied in multiple ongoing placebo-controlled outcome trials, and offer promise by virtue of the approximately 90 % reduction in CRP observed in early phase trials [27].

Current European Society of Cardiology and American College of Cardiology guidelines on acute coronary syndromes and chronic coronary disease suggest consideration of low dose colchicine to reduce MI, stroke and revascularisation [28,29]. Our findings generally reinforce these recommendations. They are also consistent with a 2025 network meta-analysis of anti-inflammatory agents that included a broader range of therapies studied in smaller early phase trials [30]. Clinical practice guidelines highlight the need for future work to identify which subgroups may derive the most benefit from colchicine therapy. Our finding that the benefits of colchicine are most pronounced in patients with established coronary artery disease are hypothesis generating and require further interrogation in collaborative meta-analyses such as those coordinated by the Colchicine Cardiovascular Trialists Collaboration [31].

The finding that some anti-inflammatory agents might increase the risk of infection has important implications. Cardiovascular disease and infection risk are biologically and epidemiologically linked by underlying immune dysregulation and inflammation, which may be especially relevant in the populations in whom anti-inflammatory therapeutics are currently being evaluated. For example, in people with chronic kidney disease, risk of atherosclerotic and non-atherosclerotic cardiovascular events increases as kidney function declines [32]. But so too does the risk of infection, with perturbations in the innate and adaptive immune system predisposing individuals with kidney disease to increased infections, virus-associated cancers, and diminished response to vaccination [33]. Indeed, infection remains the second most common cause of death after cardiovascular disease in people with chronic kidney disease, especially at advanced stages [34,35]. Similarly, for patients with heart failure, infection is one of the most common triggers for acute decompensations [36]. Our findings highlight the need to carefully evaluate this outcome in future clinical trials of anti-inflammatory agents and suggest that strategies to mitigate the risk of infection may also be important in enabling safe and effective use of these therapies.

Our results are strengthened by the systematic nature of the database search, synthesising the totality of the available randomised evidence. However, some limitations need to be considered when interpreting these results. There was limited information for several drug classes, with only a single trial for several therapeutic agents to evaluate effects on cardiovascular and safety outcomes. For some outcomes such as heart failure, there were too few events even after pooling the data to draw robust inferences about treatment effects. The lack of individual participant data precluded any assessment of the absolute effects of anti-inflammatory agents, most relevant for colchicine, across major patient groups. It also limited our ability to explore potential explanations for the observed heterogeneity across trials. Finally, the generalizability of these effect estimates may be limited by the underrepresentation of women and participants from certain regions around the world.

The impact of targeting systemic inflammation to reduce the risk of cardiovascular disease continues to be evaluated in multiple large-scale randomised, placebo-controlled outcome trials. The ZEUS trial is assessing the effect of ziltivekimab, an IL-6 inhibitor, on major adverse cardiovascular events in approximately 6200 people with chronic kidney disease, established cardiovascular disease and elevated high-sensitivity CRP, with effects on kidney disease progression also being studied [37]. In addition, ziltivekimab is being evaluated in approximately 10,000 people post myocardial infarction in the ARTEMIS trial to define the effects on major cardiovascular events [38], as well as 5600 individuals with heart failure with mildly reduced or preserved ejection fraction in the HERMES trial, with a primary outcome of hospitalisation for heart failure, urgent heart failure visit or cardiovascular death [39]. Another IL-6 inhibitor, clazakizumab, is being studied in 2200 patients with kidney failure requiring dialysis with a primary outcome of myocardial infarction or cardiovascular death [40]. These trials will better define whether a strategy of reducing systemic inflammation specifically through IL-6 inhibition can safely reduce the risk of cardiovascular events across the spectrum of cardio-kidney-metabolic conditions.

In summary, some anti-inflammatory agents may reduce the risk of cardiovascular outcomes, but their effects appear to vary substantially across drug classes, with important differences in both efficacy and safety. These results highlight the need for rigorous evaluation of new anti-inflammatory therapeutics to ascertain benefits and harms across different populations and therapeutic indications.

## Funding

Nil.

## Disclosures

**P.M. Ridker** has received institutional research grant support from Novartis, Kowa, Amarin, Pfizer, Esperion, NovoNordisk, and the NHLBI; has served as a consultant to Novartis, Flame, Agepha, AstraZeneca, Janssen, Civi Biopharm, Glaxo Smith Kline, SOCAR, Novo Nordisk, Omeicos, Health Outlook, Montai Health, New Amsterdam, Boehringer-Ingelheim, RTI; Zomagen, Cytokinetics, Horizon Therapeutics, and Cardio Therapeutics; has minority shareholder equity positions in Uppton, Bitteroot Bio, and Angiowave; and receives compensation for service on the Peter Munk Advisory Board (University of Toronto), the Leducq Foundation, Paris FR, and the Baim Institute (Boston, MA).

**K.R. Tuttle** declares receiving research grants from the National Institutes of Health, Travere Therapeutics, and the United States Centers for Disease Control and Prevention; and funding from AstraZeneca, Bayer, Boehringer Ingelheim, Eli Lilly, and Novo Nordisk.

**V. Perkovi**c serves as a Board Director for St. Vincent’s Health Australia, George Clinical and several Medical Research Institutes. He has received honoraria for Steering Committee roles, scientific presentations and/or advisory board attendance from Abbvie, Amgen, Astra Zeneca, Bayer, Baxter, Boehringer Ingelheim, Chinook, Durect, Eli Lilly, Gilead, GSK, Janssen, Merck, Mitsubishi Tanabe, Mundipharma, Novartis, Novo Nordisk, Otsuka, Pharmalink, Pfizer, Reata, Travere, Relypsa, Roche, Sanofi, Servier and Tricida.

**B.L. Neuen** has received fees for travel support, advisory boards, scientific presentations and steering committee roles from AstraZeneca, Alexion, Bayer, Boehringer and Ingelheim, Menarini, Novo Nordisk, Otsuka and Travere Therapeutics.

**A. Perkovic** and **E.R. Webster** do not have any disclosures to report.

## Data availability

The authors confirm that all data generated by this analysis is available within the manuscript and supplementary appendix.

## CRediT authorship contribution statement

**A Perkovic:** Writing – review & editing, Writing – original draft, Methodology, Formal analysis, Data curation. **ER Webster:** Writing – review & editing, Writing – original draft, Methodology, Formal analysis. **PM Ridker:** Writing – review & editing, Supervision, Methodology. **KR Tuttle:** Writing – review & editing, Supervision, Methodology. **V Perkovic:** Writing – review & editing, Writing – original draft, Supervision, Methodology, Investigation, Conceptualization. **BL Neuen:** Writing – review & editing, Writing – original draft, Supervision, Software, Methodology, Investigation.

## Declaration of competing interest

The authors declare the following financial interests/personal relationships which may be considered as potential competing interests:

Paul M Ridker reports a relationship with Novartis that includes: consulting or advisory and funding grants. Paul M Ridker reports a relationship with Kowa Company Ltd that includes: funding grants. Paul M Ridker reports a relationship with Amarin Pharma Inc that includes: funding grants. Paul M Ridker reports a relationship with Pfizer that includes: funding grants. Paul M Ridker reports a relationship with Esperion Therapeutics Inc that includes: funding grants. Paul M Ridker reports a relationship with Novo Nordisk that includes: consulting or advisory and funding grants. Paul M Ridker reports a relationship with National Heart Lung and Blood Institute that includes: funding grants. Paul M Ridker reports a relationship with FLAME that includes: consulting or advisory. Paul M Ridker reports a relationship with AGEPHA that includes: consulting or advisory. Paul M Ridker reports a relationship with AstraZeneca Pharmaceuticals LP that includes: consulting or advisory. Paul M Ridker reports a relationship with Janssen Pharmaceuticals Inc that includes: consulting or advisory. Paul M Ridker reports a relationship with CiVi Biopharma Inc that includes: consulting or advisory. Paul M Ridker reports a relationship with GLAXO SMITHKLINE PTY LTD EMC that includes: consulting or advisory. Paul M Ridker reports a relationship with SOCAR that includes: consulting or advisory. Paul M Ridker reports a relationship with OMEICOS Therapeutics GmbH that includes: consulting or advisory. Paul M Ridker reports a relationship with Health Outlook that includes: consulting or advisory. Paul M Ridker reports a relationship with Montai Therapeutics Inc that includes: consulting or advisory. Paul M Ridker reports a relationship with New Amsterdam that includes: consulting or advisory. Paul M Ridker reports a relationship with Boehringer Ingelheim Pharmaceuticals Inc that includes: consulting or advisory. Paul M Ridker reports a relationship with RTI that includes: consulting or advisory. Paul M Ridker reports a relationship with ZOMAGEN that includes: consulting or advisory. Paul M Ridker reports a relationship with Cytokinetics Inc that includes: consulting or advisory. Paul M Ridker reports a relationship with Horizon Therapeutics plc that includes: consulting or advisory. Paul M Ridker reports a relationship with CARDIO THERAPEUTICS that includes: consulting or advisory. Paul M Ridker reports a relationship with UPPTON that includes: equity or stocks. Paul M Ridker reports a relationship with BITTEROOT BIO that includes: equity or stocks. Paul M Ridker reports a relationship with ANGIOWAVE that includes: equity or stocks. Paul M Ridker reports a relationship with Peter Munk Cardiac Centre that includes: consulting or advisory. Paul M Ridker reports a relationship with Leducq Foundation that includes: consulting or advisory. Paul M Ridker reports a relationship with PARIS FR that includes: consulting or advisory. Paul M Ridker reports a relationship with Baim Institute for Clinical Research that includes: consulting or advisory. Katherine R Tuttle reports a relationship with National Institutes of Health that includes: funding grants. Katherine R Tuttle reports a relationship with Travere Therapeutics Inc that includes: funding grants. Katherine R Tuttle reports a relationship with Centers for Disease Control and Prevention that includes: funding grants. Katherine R Tuttle reports a relationship with AstraZeneca Pharmaceuticals LP that includes: funding grants. Katherine R Tuttle reports a relationship with Bayer Corporation that includes: funding grants. Katherine R Tuttle reports a relationship with Boehringer Ingelheim Pharmaceuticals Inc that includes: funding grants. Katherine R Tuttle reports a relationship with Eli Lilly and Company that includes: funding grants. Katherine R Tuttle reports a relationship with Novo Nordisk Inc that includes: funding grants. Vlado Perkovic reports a relationship with St Vincent’s Health Australia Ltd that includes: board membership. Vlado Perkovic reports a relationship with George Clinical Pty Ltd that includes: board membership. Vlado Perkovic reports a relationship with AbbVie Inc that includes: consulting or advisory. Vlado Perkovic reports a relationship with Amgen Inc that includes: consulting or advisory. Vlado Perkovic reports a relationship with AstraZeneca Pharmaceuticals LP that includes: consulting or advisory. Vlado Perkovic reports a relationship with Bayer Corporation that includes: consulting or advisory. Vlado Perkovic reports a relationship with Baxter International Inc that includes: consulting or advisory. Vlado Perkovic reports a relationship with Boehringer Ingelheim Pharmaceuticals Inc that includes: consulting or advisory. Vlado Perkovic reports a relationship with Chinook Therapeutics Inc that includes: consulting or advisory. Vlado Perkovic reports a relationship with Durect Corporation that includes: consulting or advisory. Vlado Perkovic reports a relationship with Eli Lilly that includes: consulting or advisory. Vlado Perkovic reports a relationship with Gilead Sciences Inc that includes: consulting or advisory. Vlado Perkovic reports a relationship with GSK that includes: consulting or advisory. Vlado Perkovic reports a relationship with Janssen Pharmaceuticals Inc that includes: consulting or advisory. Vlado Perkovic reports a relationship with Merck & Co Inc that includes: consulting or advisory. Vlado Perkovic reports a relationship with Mitsubishi Tanabe Pharma Corporation that includes: consulting or advisory. Vlado Perkovic reports a relationship with Mundipharma Pty Ltd that includes: consulting or advisory. Vlado Perkovic reports a relationship with Novartis Pharmaceuticals Corporation that includes: consulting or advisory. Vlado Perkovic reports a relationship with Novo Nordisk Inc that includes: consulting or advisory. Vlado Perkovic reports a relationship with Otsuka Pharmaceutical Co Ltd that includes: consulting or advisory. Vlado Perkovic reports a relationship with PharmaLink Inc that includes: consulting or advisory. Vlado Perkovic reports a relationship with Pfizer that includes: consulting or advisory. Vlado Perkovic reports a relationship with Reata Pharmaceuticals Inc that includes: consulting or advisory. Vlado Perkovic reports a relationship with Travere Therapeutics Inc that includes: consulting or advisory. Vlado Perkovic reports a relationship with RELYPSA that includes: consulting or advisory. Vlado Perkovic reports a relationship with Roche that includes: consulting or advisory. Vlado Perkovic reports a relationship with Sanofi that includes: consulting or advisory. Vlado Perkovic reports a relationship with SERVIER that includes: consulting or advisory. Vlado Perkovic reports a relationship with Tricida Inc that includes: consulting or advisory. Brendon L Neuen reports a relationship with AstraZeneca Pharmaceuticals LP that includes: consulting or advisory. Brendon L Neuen reports a relationship with Alexion that includes: consulting or advisory. Brendon L Neuen reports a relationship with Bayer Corporation that includes: consulting or advisory. Brendon L Neuen reports a relationship with Boehringer Ingelheim Pharmaceuticals Inc that includes: consulting or advisory. Brendon L Neuen reports a relationship with Menarini Laboratories that includes:. Brendon L Neuen reports a relationship with Novo Nordisk that includes: consulting or advisory. Brendon L Neuen reports a relationship with Otsuka Pharmaceutical Co Ltd that includes: consulting or advisory. Brendon L Neuen reports a relationship with Travere Therapeutics Inc that includes: consulting or advisory. If there are other authors, they declare that they have no known competing financial interests or personal relationships that could have appeared to influence the work reported in this paper.

## Appendix Supplementary materials

Image, application 1
