# Supplementary material for: Effects of anti-inflammatory agents on cardiovascular outcomes: A systematic review and meta-analysis of randomised controlled trials
Source: Am J Prev Cardiol. 2026 Apr 17;27:101642. doi: 10.1016/j.ajpc.2026.101642 (PMC13261216; doi:10.1016/j.ajpc.2026.101642)

**SUPPLEMENTARY PUBLICATION MATERIAL**

Table of Contents

Table S1. Search strategy………………………………………………………………………….2

Figure S1. PRISMA diagram………………………………………………………………………5

Figure S2. Risk of bias assessment………………………………………………………………...6

Figure S3. Funnel plot for publication bias………………………………………………………...7

Figure S4. Sensitivity analysis for the primary outcome excluding non-coronary artery disease and/or post-myocardial infarction trials…………………………………………………………...8

Figure S5. Sensitivity analysis for the primary outcome excluding trials in which all-cause death was included as part of the MACE composite outcome…………………………………………...9

Figure S6. MI..……………………………………………………………….………………......10

Figure S7. Stroke.…………………………………………………………… ………………......11

Figure S8. HHF.…………………………………………………………….. ……………….......12

Figure S9. CV death..…………………….………..……………………….. ……………….......13

Figure S10. ACM….……………………………………………………….....……………….....14

Figure S11. Adverse events...………………………………………………...………………......15

Figure S12. Cancer.………..……………………………………………........………………......16

Figure S13. Infection.………………………………………………………...………………......17

**Table S1: search strategy.**

| Medline and Embase via Ovid   1. Exp Randomized Controlled Trial/ 2. Exp Random Allocation/ 3. Exp Single-Blind Method/ 4. Exp Double-Blind Method/ 5. (random$ adj5 trial$).tw. 6. (random$ adj5 allocation$).tw. 7. (Blind$ adj5 method$).tw. 8. 1 OR 2 OR 3 OR 4 OR 5 OR 6 OR 7 9. Exp Mortality/ 10. Exp Cardiovascular diseases/ 11. Exp Heart failure/ 12. Cardiovascular death.tw. 13. Exp Myocardial infarction/ 14. Exp Stroke/ 15. Exp Coronary disease/ 16. Revasculari?ation.tw. 17. Cardiovascular outcome$.tw. 18. 9 OR 10 OR 11 OR 12 OR 13 OR 14 OR 15 OR 16 OR 17 19. Exp Methotrexate/ 20. Exp Folic Acid Antagonists/ 21. Exp Colchicine/ 22. Interleukin 1 inhibit$.tw. 23. Interleukin 1 antagonis$.tw. 24. IL 1? inhibit$.tw. 25. IL 1? antagonis$.tw. 26. IL1? inhibit$.tw. 27. IL1? antagonis$.tw. 28. Canakinumab.tw. 29. Anakinra.tw. 30. Rilonacept.tw. 31. Interleukin 6 inhibit$.tw. 32. Interleukin 6 antagonis$.tw. 33. IL 6 inhibit$.tw. 34. IL 6 antagonis$.tw. 35. IL6 inhibit$.tw. 36. IL6 antagonis$.tw. 37. Toclizumab.tw. 38. Sarilumab.tw. 39. Satralizumab.tw. 40. Siltuximab.tw. 41. Ziltivekimab.tw. 42. Exp Janus Kinase Inhibitors/ 43. Baricitinib.tw. 44. Tofacitinib.tw. 45. Upadacitinib.tw. 46. Nuclear factor erythroid 2-related factor 2 activat$.tw. 47. NRF 2 activat$.tw. 48. NRF2 activat$.tw. 49. Bardoxolone.tw. 50. ASK 1 inhibit$.tw. 51. ASK1 inhibit$.tw. 52. ASK 1 antagonis$.tw. 53. ASK1 antagonis$.tw. 54. Apoptosis signal-regulating kinase 1 inhibit$.tw. 55. Apoptosis signal-regulating kinase 1 antagonis$.tw. 56. Selonsertib.tw. 57. Protein kinase C alpha inhibit$.tw. 58. Protein kinase C alpha antagonis$.tw. 59. PKC-alpha inhibit$.tw. 60. PKC-alpha antagonis$.tw. 61. Ruboxistaurin.tw. 62. C-C chemokine receptor type 2 inhibit$.tw. 63. C-C chemokine receptor type 2 antagonis$.tw. 64. C-C chemokine ligand type 2 inhibit$.tw. 65. C-C chemokine ligand type 2 antagonis$.tw. 66. (CC$ adj4 antagonis$).tw. 67. (CC$ adj4 inhibit$).tw. 68. (CC$ adj3 antagonis$).tw. 69. (CC$ adj3 inhibit$).tw. 70. Bindarit.tw. 71. CCX140-B.tw. 72. Darapladib.tw. 73. Phospholipase A2 inhibit$.tw. 74. Phospholipase A2 antagonis$.tw. 75. PLA2 inhibit$.tw. 76. PLA2 antagonis$.tw. 77. 19 OR 20 OR 21 OR 22 OR 23 OR 24 OR 25 OR 26 OR 27 OR 28 OR 29 OR 30 OR 31 OR 32 OR 33 OR 34 OR 35 OR 36 OR 37 OR 38 OR 39 OR 40 OR 41 OR 42 OR 43 OR 44 OR 45 OR 46 OR 47 OR 48 OR 49 OR 50 OR 51 OR 52 OR 53 OR 54 OR 55 OR 56 OR 57 OR 58 OR 59 OR 60 OR 61 OR 62 OR 63 OR 64 OR 65 OR 66 OR 67 OR 68 OR 69 OR 70 OR 71 OR 72 OR 73 OR 74 OR 75 OR 76 78. 8 AND 18 AND 77 | The Cochrane Library   1. Randomized Controlled Trial 2. Random Allocation 3. Single-Blind Method 4. Double-Blind Method 5. Random* trial*:ti,ab,kw 6. Random* allocation*:ti,ab,kw 7. Blind* method*:ti,ab,kw 8. 1 OR 2 OR 3 OR 4 OR 5 OR 6 OR 7 9. Mortality 10. Cardiovascular diseases 11. Heart failure 12. Cardiovascular death:ti,ab,kw 13. Myocardial infarction 14. Stroke 15. Coronary disease 16. Revasculari?ation:ti,ab,kw 17. Cardiovascular outcome*:ti,ab,kw 18. 9 OR 10 OR 11 OR 12 OR 13 OR 14 OR 15 OR 16 OR 17 19. Methotrexate 20. Folic Acid Antagonists 21. Colchicine 22. Interleukin 1 inhibit*:ti,ab,kw 23. Interleukin 1 antagonis*:ti,ab,kw 24. IL 1? Inhibit*:ti,ab,kw 25. IL 1? Antagonis*:ti,ab,kw 26. IL1? Inhibit*:ti,ab,kw 27. IL1? Antagonis*:ti,ab,kw 28. Canakinumab:ti,ab,kw 29. Anakinra:ti,ab,kw 30. Rilonacept:ti,ab,kw 31. Interleukin 6 inhibit*:ti,ab,kw 32. Interleukin 6 antagonis*:ti,ab,kw 33. IL 6 inhibit*:ti,ab,kw 34. IL 6 antagonis*:ti,ab,kw 35. IL6 inhibit*:ti,ab,kw 36. IL6 antagonis*:ti,ab,kw 37. Toclizumab:ti,ab,kw 38. Sarilumab:ti,ab,kw 39. Satralizumab:ti,ab,kw 40. Siltuximab:ti,ab,kw 41. Ziltivekimab:ti,ab,kw 42. Janus Kinase Inhibitors 43. Baricitinib:ti,ab,kw 44. Tofacitinib:ti,ab,kw 45. Upadacitinib:ti,ab,kw 46. Nuclear factor erythroid 2-related factor 2 activat*:ti,ab,kw 47. NRF 2 activat*:ti,ab,kw 48. NRF2 activat*:ti,ab,kw 49. Bardoxolone:ti,ab,kw 50. ASK 1 inhibit*:ti,ab,kw 51. ASK1 inhibit*:ti,ab,kw 52. ASK 1 antagonis*:ti,ab,kw 53. ASK1 antagonis*:ti,ab,kw 54. Apoptosis signal-regulating kinase 1 inhibit*:ti,ab,kw 55. Apoptosis signal-regulating kinase 1 antagonis*:ti,ab,kw 56. Selonsertib:ti,ab,kw 57. Protein kinase C alpha inhibit*:ti,ab,kw 58. Protein kinase C alpha antagonis*:ti,ab,kw 59. PKC-alpha inhibit*:ti,ab,kw 60. PKC-alpha antagonis*:ti,ab,kw 61. Ruboxistaurin:ti,ab,kw 62. C?C chemokine receptor type 2 inhibit*:ti,ab,kw 63. C?C chemokine receptor type 2 antagonis*:ti,ab,kw 64. C?C chemokine ligand type 2 inhibit*:ti,ab,kw 65. C?C chemokine ligand type 2 antagonis*:ti,ab,kw 66. (CC* antagonis*):ti,ab,kw 67. (CC* inhibit*):ti,ab,kw 68. Bindarit:ti,ab,kw 69. CCX140?B:ti,ab,kw 70. Darapladib:ti,ab,kw 71. Phospholipase A2 inhibit*:ti,ab,kw 72. Phospholipase A2 antagonis*:ti,ab,kw 73. PLA2 inhibit*:ti,ab,kw 74. PLA2 antagonis*:ti,ab,kw 75. 19 OR 20 OR 21 OR 22 OR 23 OR 24 OR 25 OR 26 OR 27 OR 28 OR 29 OR 30 OR 31 OR 32 OR 33 OR 34 OR 35 OR 36 OR 37 OR 38 OR 39 OR 40 OR 41 OR 42 OR 43 OR 44 OR 45 OR 46 OR 47 OR 48 OR 49 OR 50 OR 51 OR 52 OR 53 OR 54 OR 55 OR 56 OR 57 OR 58 OR 59 OR 60 OR 61 OR 62 OR 63 OR 64 OR 65 OR 66 OR 67 OR 68 OR 69 OR 70 OR 71 OR 72 OR 73 OR 74 76. 8 AND 18 AND 75 |
| --- | --- |

**Figure S1: PRISMA diagram.**

**Identification of studies via databases and registers**

Records removed *before screening*:

Duplicate records removed (n = 1167)

Records identified from Medline, Embase and Cochrane databases:

(n = 10353)

Medline (n = 1081)

Embase (n = 6745)

Cochrane (n = 2527)

**Identification**

Records screened by title and abstract

(n = 9186)

Records excluded

(n = 8708)

Reports sought for retrieval

(n = 478)

Reports not retrieved

(n = 0)

**Screening**

Reports assessed for eligibility

(n = 478)

Reports excluded: (n = 356)

Background anti-inflammatories (n = 142)

Insufficient size (n = 103)

Design not RCT (n = 58)

Incorrect population (n = 9)

Incorrect intervention (n = 2)

Outcome (n=42)

Studies included in review

(n = 13)

Reports of included studies

(n = 122)

**Included**

**Figure S2: Cochrane Risk of Bias 2 Tool (RoB2).**


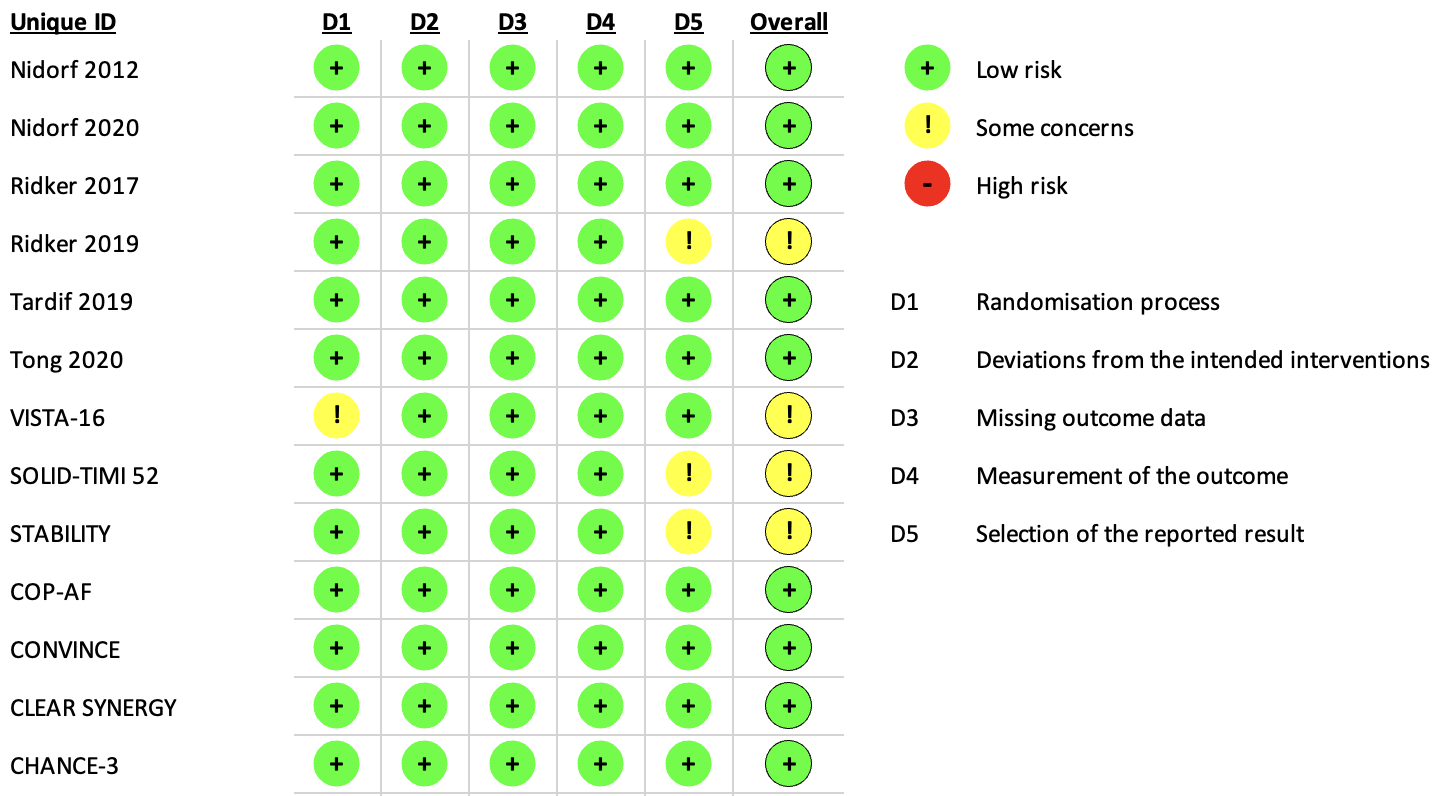


**Figure S3: Funnel plot evaluating small-study effects and potential publication bias among included trials.**

Funnel plot displaying log-transformed hazard ratios and corresponding standard errors for included trials. Each point represents a single study. The vertical line denotes the pooled inverse-variance estimate, and the diagonal lines indicate pseudo–95% confidence limits used to visually assess potential publication bias.

**Figure S4: Sensitivity analysis for the primary outcome excluding non-coronary artery disease and/or post myocardial infarction trials.**

**Figure S5: Sensitivity analysis for the primary outcome excluding trials in which all-cause death was included as part of the MACE composite outcome.**

**Figure S6:** **Effect of anti-inflammatory agents on myocardial infarction.**


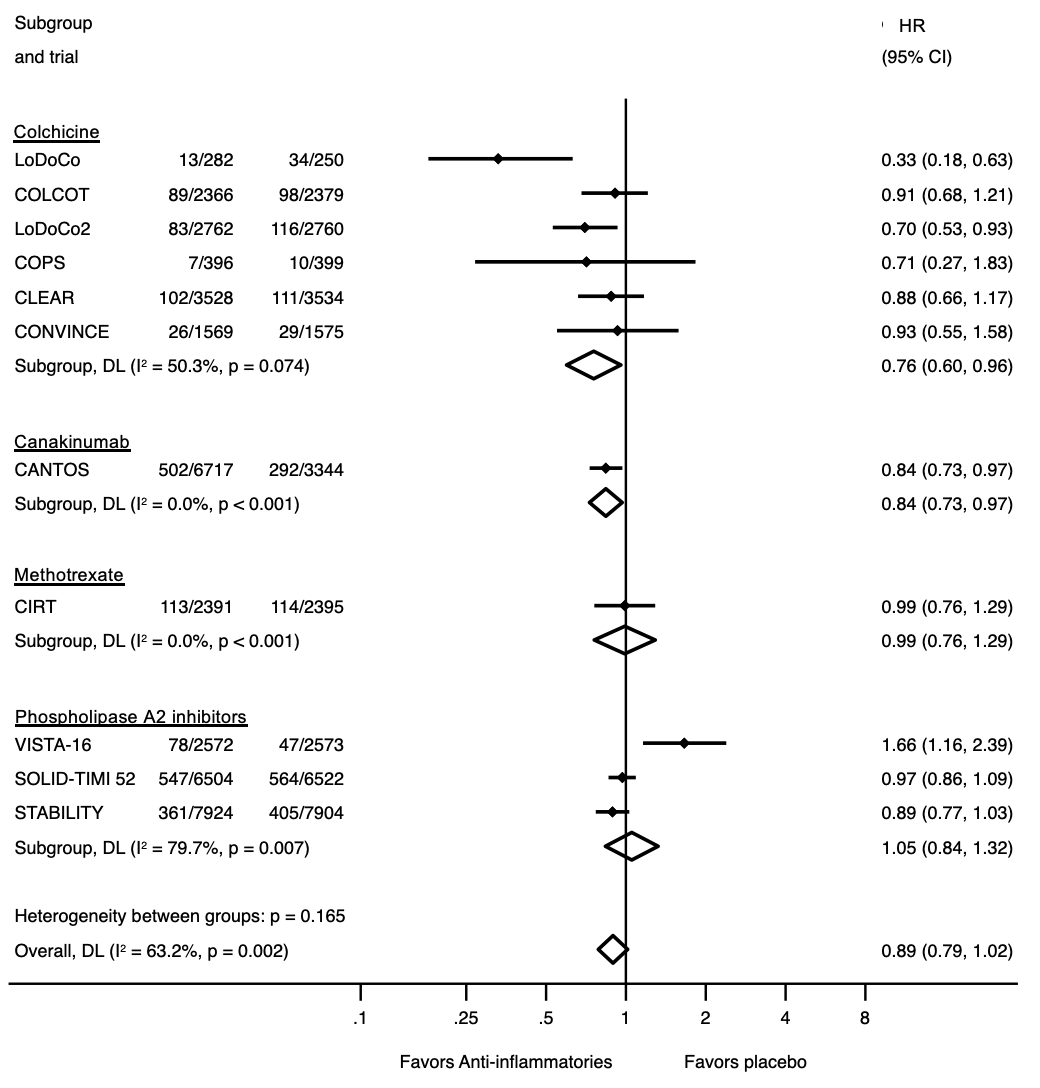


**Figure S7:** **Effect of anti-inflammatory agents on stroke.**


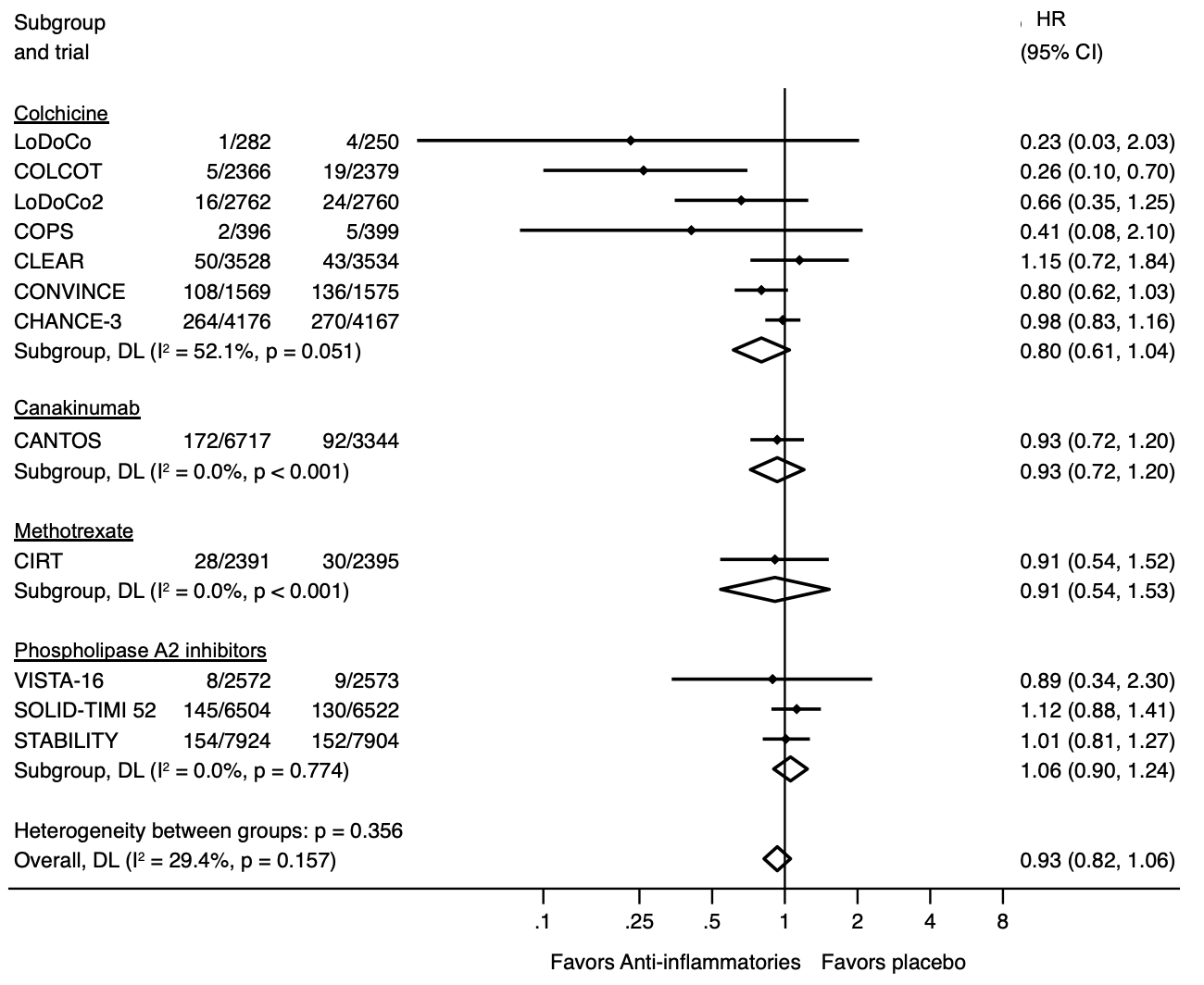


**Figure S8:** **Effect of anti-inflammatory agents on hospitalization for heart failure.**


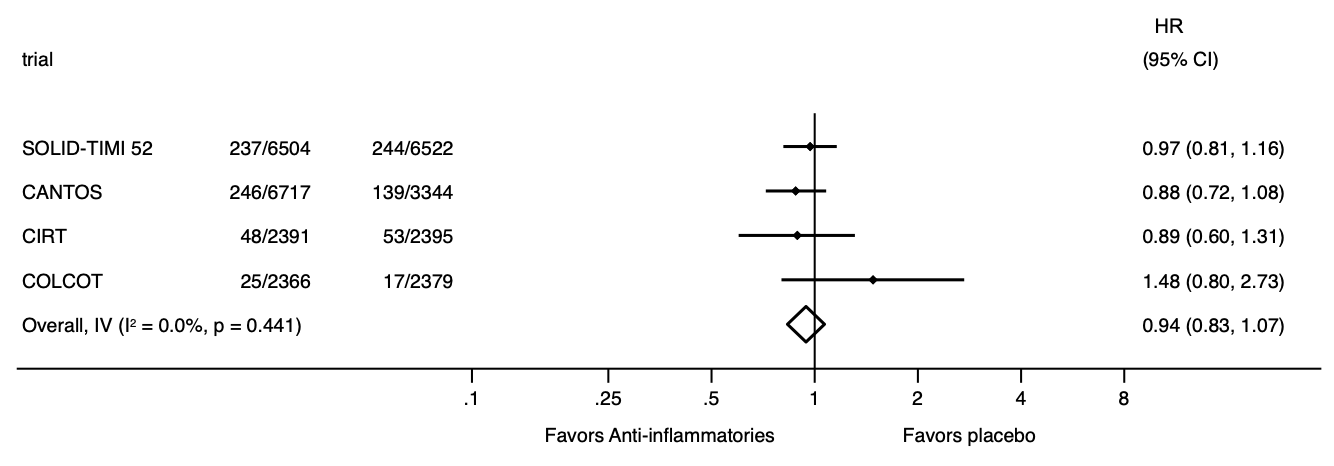


**Figure S9:** **Effect of anti-inflammatory agents on cardiovascular death.**


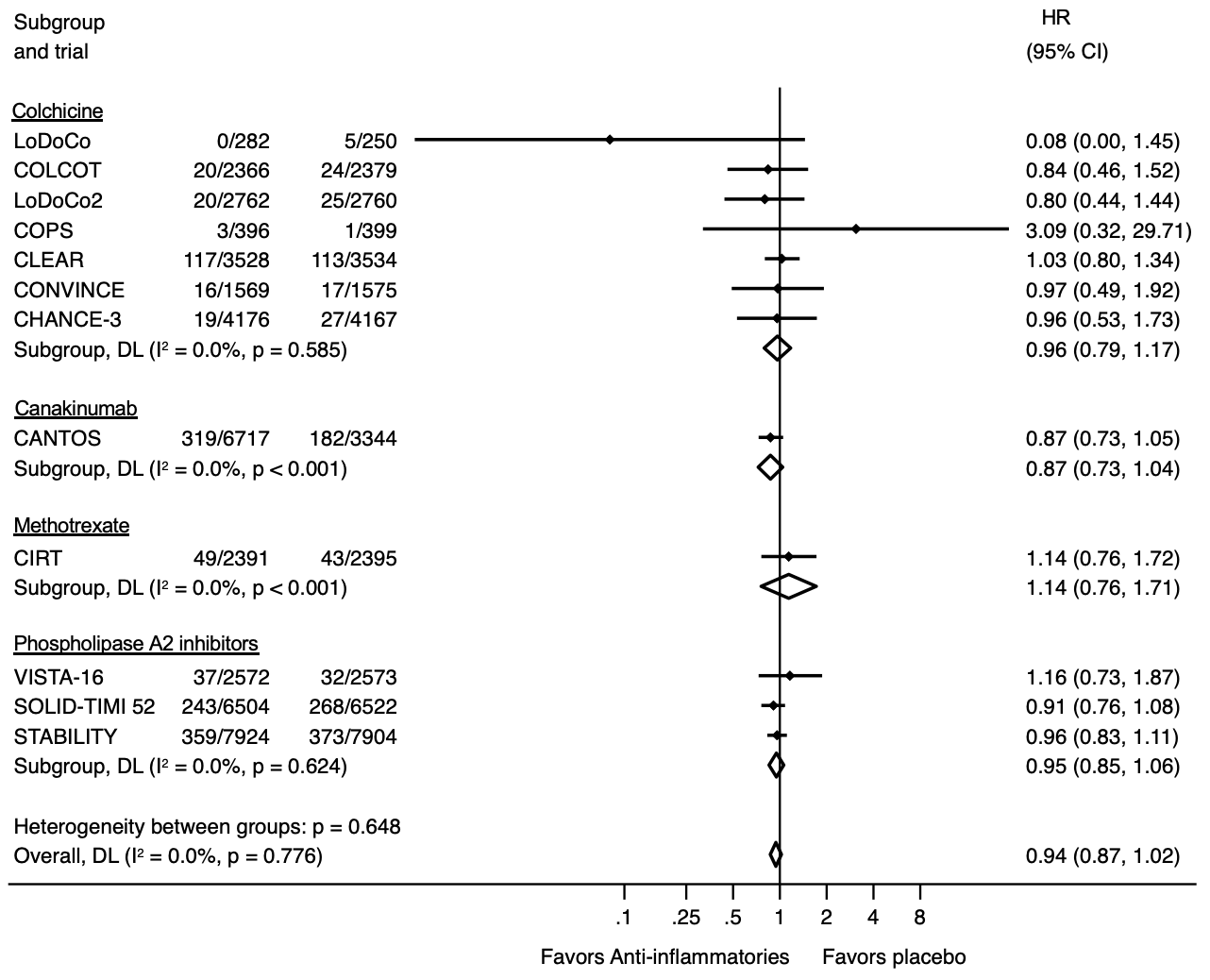


**Figure S10:** **Effect of anti-inflammatory agents on all-cause mortality.**


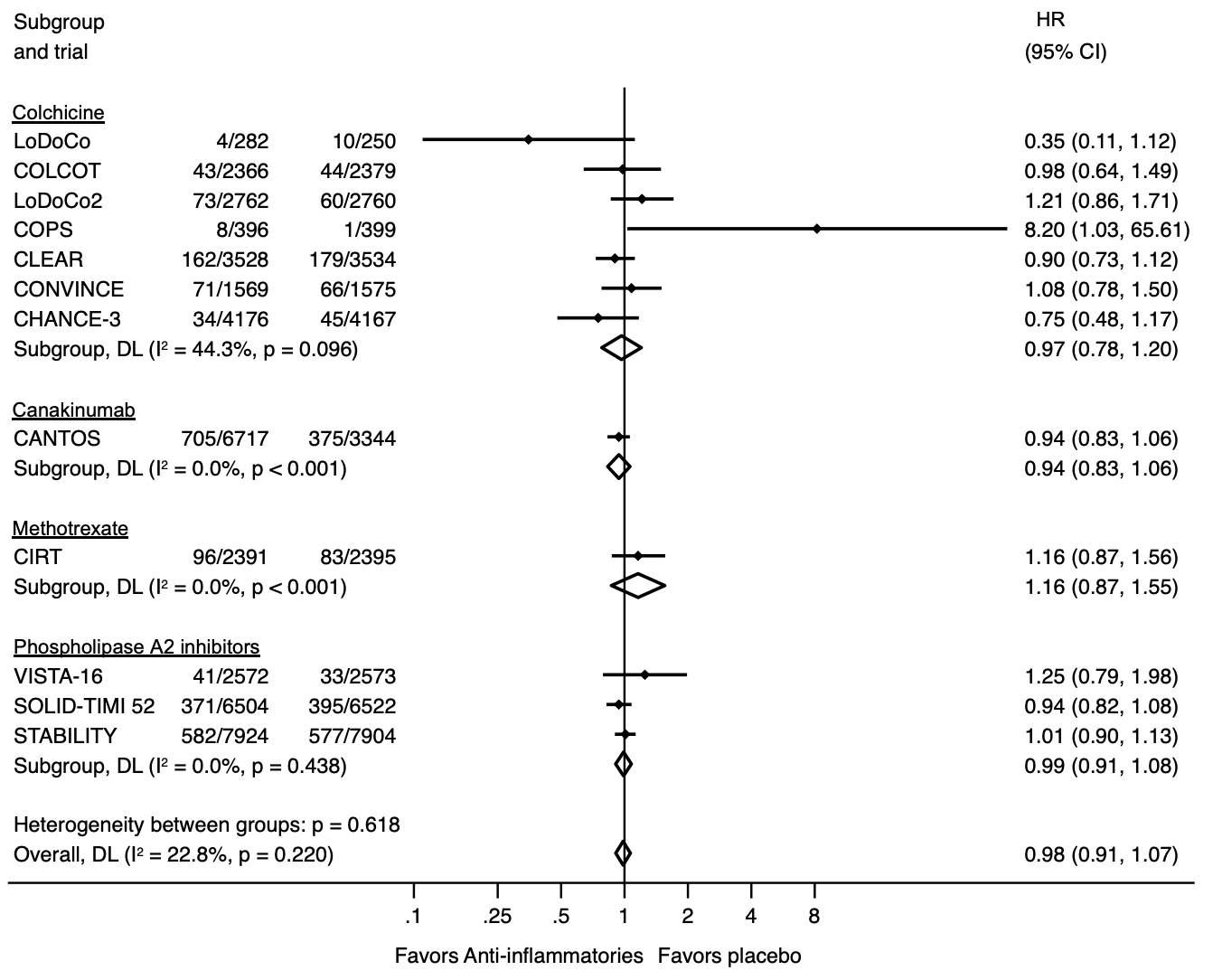


**Figure S11:** **Effect of anti-inflammatory agents on serious adverse effects.**


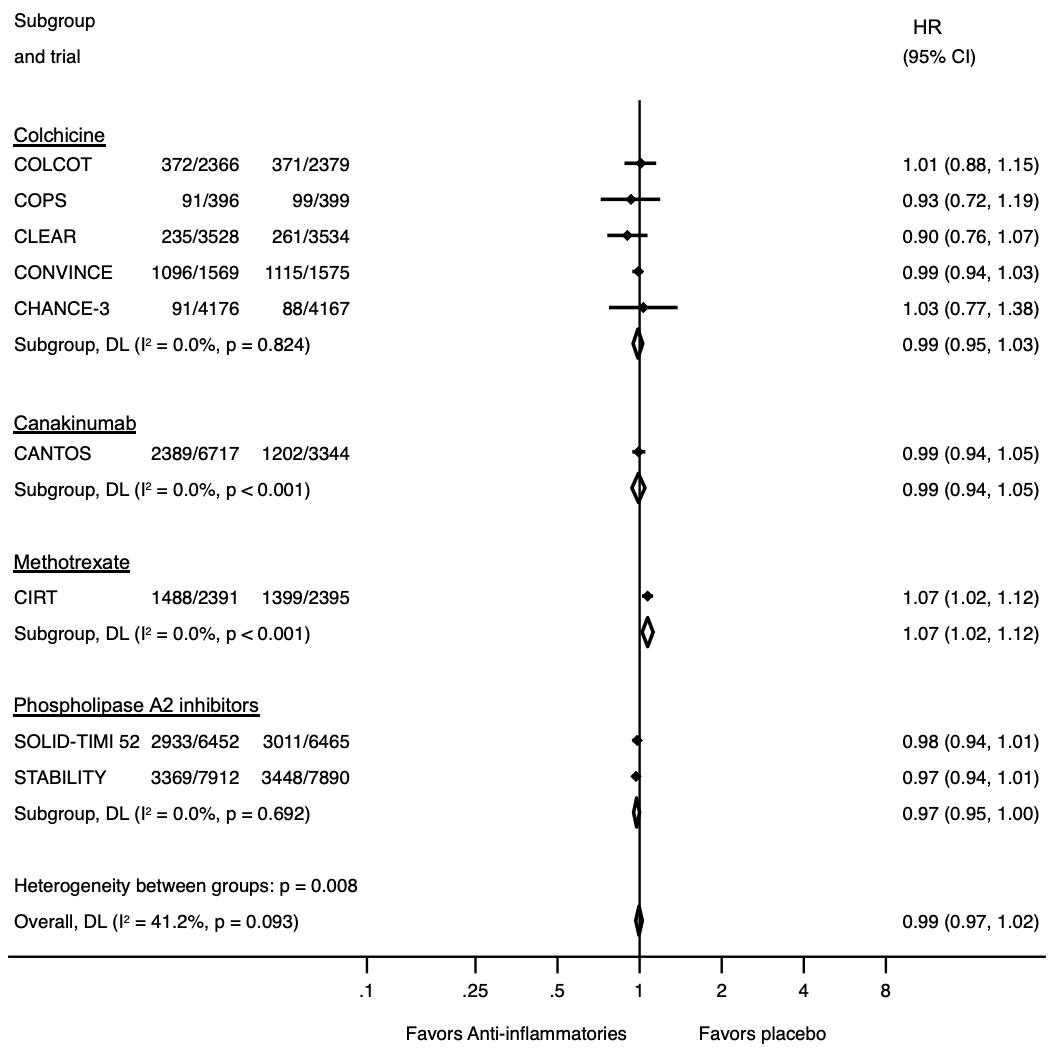


**Figure S12:** **Effect of anti-inflammatory agents on cancer incidence.**


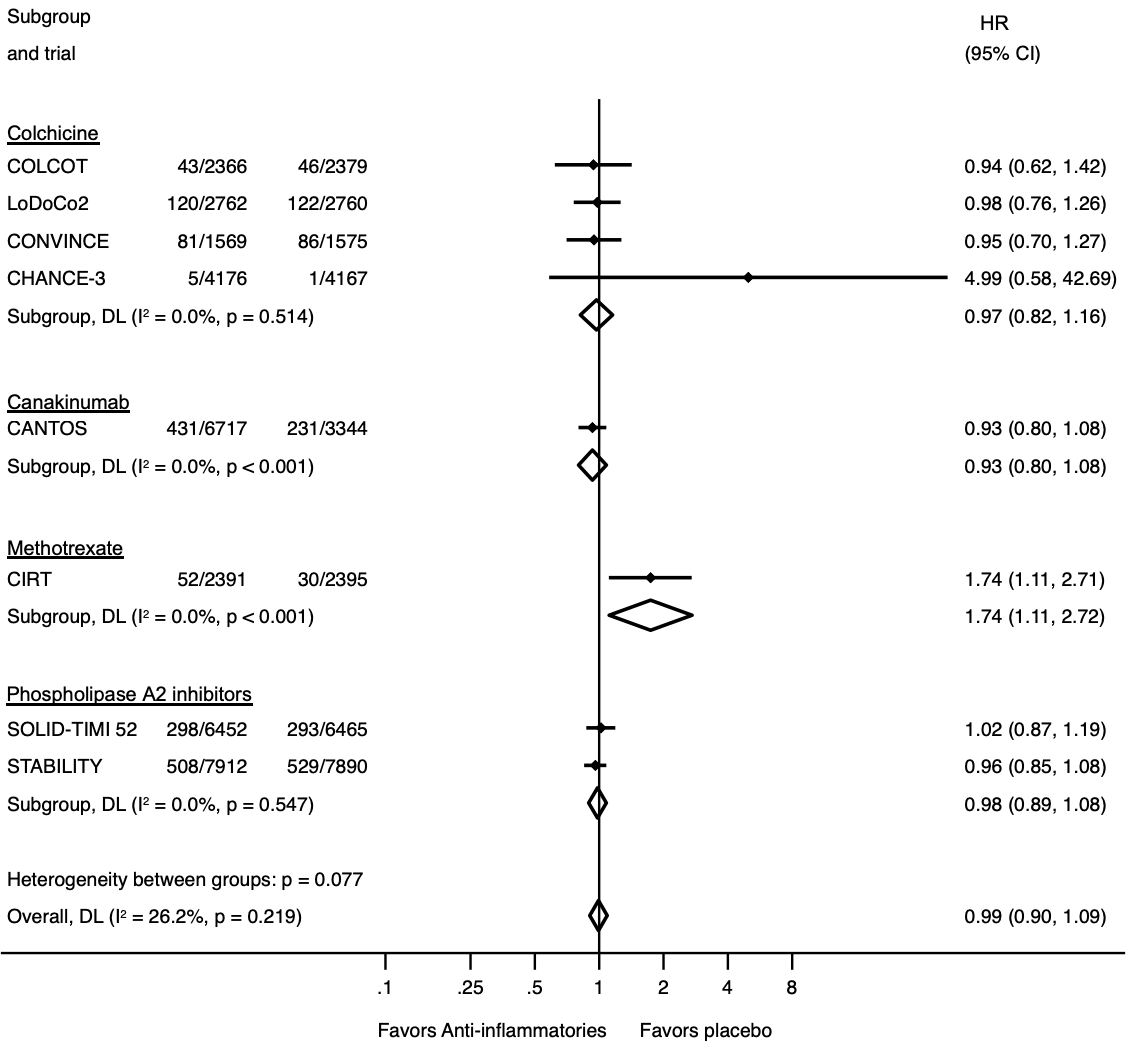


**Figure S13:** **Effect of anti-inflammatory agents on infection.**


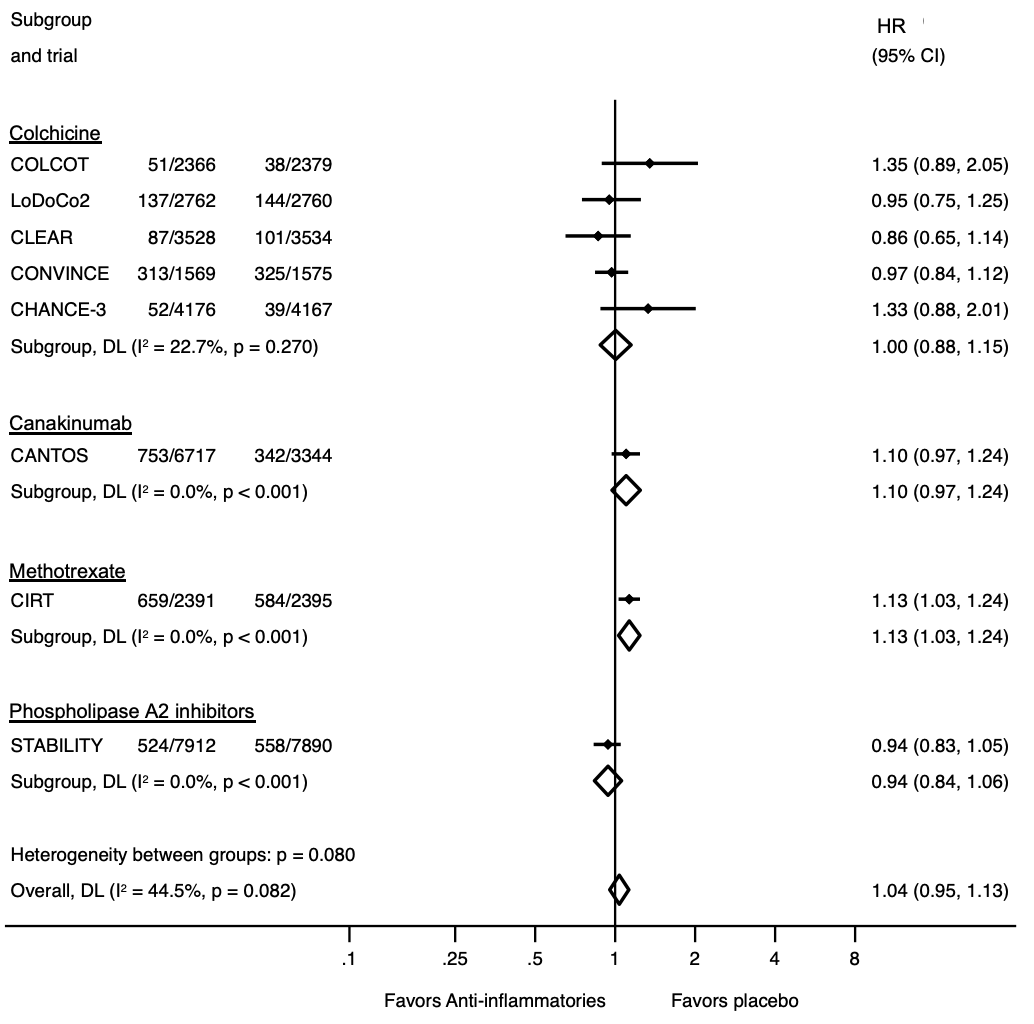

Supplement: Supplementary file 1 [file mmc1.docx]
